# The Bright Side of Psychedelics: Latest Advances and Challenges in Neuropharmacology

**DOI:** 10.3390/ijms24021329

**Published:** 2023-01-10

**Authors:** Andrea Mastinu, Margrate Anyanwu, Marinella Carone, Giulia Abate, Sara Anna Bonini, Gregorio Peron, Emanuela Tirelli, Mariachiara Pucci, Giovanni Ribaudo, Erika Oselladore, Marika Premoli, Alessandra Gianoncelli, Daniela Letizia Uberti, Maurizio Memo

**Affiliations:** Department of Molecular and Translational Medicine, University of Brescia, Viale Europa 11, 25123 Brescia, Italy

**Keywords:** psychedelics, ibogaine, mescaline, N,N-dimethyltryptamine, psilocybin, psilocin, lysergic acid diethylamide, molecular docking, psychedelic assisted psychotherapy

## Abstract

The need to identify effective therapies for the treatment of psychiatric disorders is a particularly important issue in modern societies. In addition, difficulties in finding new drugs have led pharmacologists to review and re-evaluate some past molecules, including psychedelics. For several years there has been growing interest among psychotherapists in psilocybin or lysergic acid diethylamide for the treatment of obsessive-compulsive disorder, of depression, or of post-traumatic stress disorder, although results are not always clear and definitive. In fact, the mechanisms of action of psychedelics are not yet fully understood and some molecular aspects have yet to be well defined. Thus, this review aims to summarize the ethnobotanical uses of the best-known psychedelic plants and the pharmacological mechanisms of the main active ingredients they contain. Furthermore, an up-to-date overview of structural and computational studies performed to evaluate the affinity and binding modes to biologically relevant receptors of ibogaine, mescaline, N,N-dimethyltryptamine, psilocin, and lysergic acid diethylamide is presented. Finally, the most recent clinical studies evaluating the efficacy of psychedelic molecules in some psychiatric disorders are discussed and compared with drugs already used in therapy.

## 1. Introduction

In both the past and in modern human primitive societies, psychedelic molecules have been used to alter a shaman’s state of consciousness and put them in contact with divinity [1,2,3]. Shamans are well acquainted with nature and particularly with some of the therapeutic effects of plants and mushrooms. Therefore, in addition to performing a religious function, they are also considered healers [1]. The knowledge of the sacredness of nature and the therapeutic virtues of plants and fungi are the basis of the concepts of “entheogen” and “teacher plant” [1,4,5]. Entheogens are those molecules that are derived from plants and/or fungi that alter the perception of space and time and states of consciousness, allowing the user to communicate with the divinity or the dead [6]. The concept of “teacher plants” is based on the plants’ ability to transmit their “therapeutic knowledge” to the shaman who ingests, or smokes, the plant itself [7]. These concepts were developed by the first anthropologists of the last century who described shamanic practices during their travels among the Amazonian Indians, American Indians, or among the people of Central Africa [8,9,10,11]. Since the 1960s, the use of plant derivatives based on psychedelic molecules has also spread to Western countries [12,13]. Based on the chronicles of those years, artists and intellectuals were fascinated by psychedelic molecules that allowed them to “think outside the box”, and, therefore, increase their creativity [14,15,16,17,18]. In the following years, governments imposed strict bans on the use of psychedelics, considering them to be narcotics [19]. These restrictions have hampered pharmaceutical research aimed at understanding the neuronal molecular mechanisms underlying the response of psychedelics [19].

However, in the last decade, psychedelic-assisted psychotherapy (PAP) has emerged as an alternative strategy, one that arose in response to the crisis of new psychiatric drugs [20,21,22,23,24]. To date, there have been promising clinical trials of PAP with LSD, psilocybin and ibogaine to combat drug, alcohol and nicotine addiction [25,26,27,28]. Recently, potential applications have been found in the treatment of anxiety, obsessive compulsive disorders, major depression, autism spectrum disorders, and, finally, in delaying cognitive decline [27,29,30,31,32,33].

**Figure 1:**
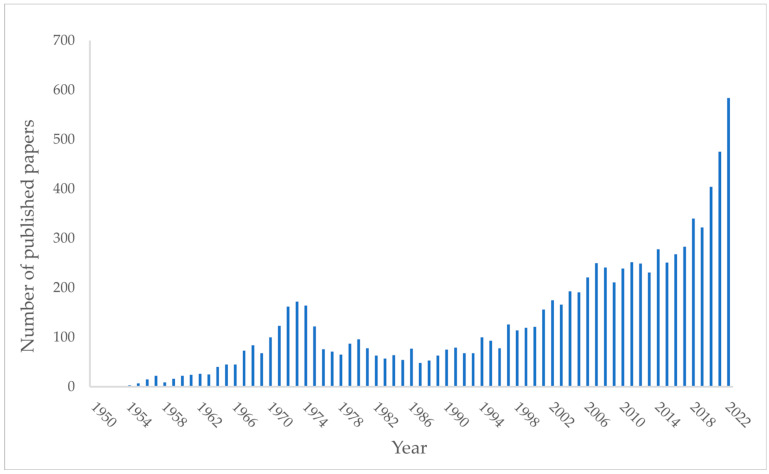
Number of papers published per year from 1952 to 2022 reported on PubMed (https://pubmed.ncbi.nlm.nih.gov, accessed on 13 December 2022) by searching for “psychedelic therapies”. Although the research on this topic has flourished increasingly throughout the years, a decrease in the number of papers can be observed in the early 70s. In that period, research on psychedelic drugs was partially abandoned for several reasons, including tighter regulations connected to Richard Nixon’s ‘War on Drugs’ [34].

This study was designed to give a rationale for PAP. If we search the words “psychedelic therapies” on PubMed (Figure 1), the number of papers in this field published in the past 10 years has increased (from 249 papers in 2012 to 582 papers in 2022).

This review aims to point out the therapeutic potential of *Tabernanthe iboga*, *Echinocactus williamsii*, *Psychotria viridis*, *Psilocybe cubensis*, and *Claviceps purpurea*. The choice of these plant and fungi species is related to the applications of their main active ingredients in psychedelic-assisted psychotherapy.

Emphasis will be placed on the molecular mechanisms that determine the psychedelic response. To this purpose, structural and computational studies investigating the interaction of psychedelic molecules with their macromolecular targets have been considered for the preparation of this article.

In this regard, 3D structures reported in the current review were produced using UCSF Chimera.

## 2. Tabernanthe Iboga

### 2.1. Ethnobotany

**Figure 2:**
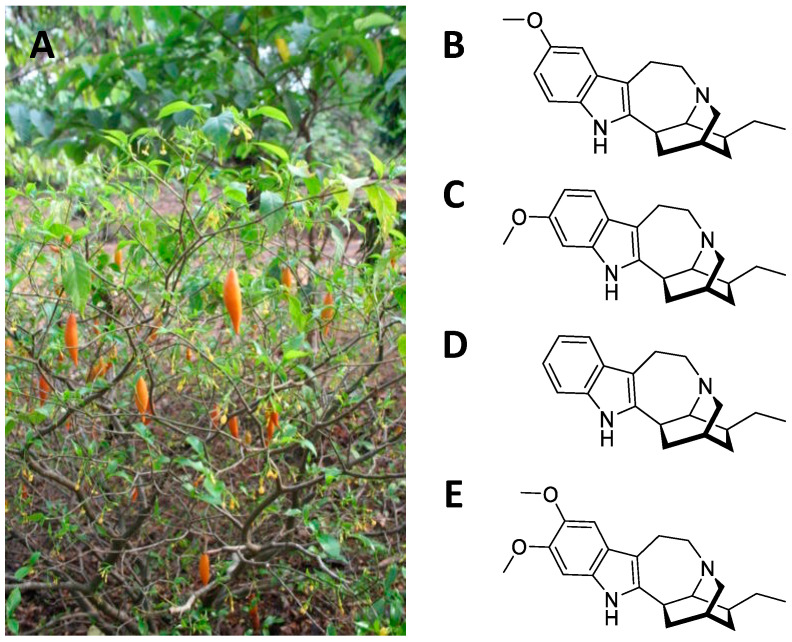
*Tabernanthe iboga* with orange fruits (**A**); chemical structures of ibogaine (**B**); tabernanthine (**C**); ibogamine (**D**); ibogaline (**E**).

*Tabernanthe iboga* is a shrub from equatorial Africa belonging to the Apocinaceae family (Figure 2A). The root is rich in monomeric polycyclic indole alkaloids (1–3% in the whole root, 5–6% in the bark), the primary one being ibogaine, followed by tabernantin, ibogamine, and ibogalin (Figure 2B–E) [35]. The root was used by the African natives as a nervous stimulant (to deal with hunger and fatigue) [8]. In addition, it was also used as an aphrodisiac, due to the transient excitement it produces [36]. These properties are confirmed in the current uses of the plant as a neuro-muscular tonic, appetite stimulant, anti-asthenic, and antidepressant [37]. The psychedelic and hallucinatory action is mainly a result of the ibogaine [35]. This alkaloid causes visual hallucinations, often associated with a strong state of anxiety and apprehension [38]. At toxic doses, convulsions, bradycardia, hypotension, paralysis, and respiratory arrest occur [38].

### 2.2. Central Nervous System Pathways

Over the years, numerous pharmacological studies on the effects of ibogaine and its derivatives have been reported. Early research examined the effects of ibogaine on the central nervous system and on the cardiovascular system [39]. Lambarene^®^ was an ibogaine-based drug marketed in France as a “neurostimulant” [39]. However, at the end of the 1960s the drug was withdrawn and ibogaine was included among the doping substances in sports and was made illegal in almost all countries [40]. Despite the prohibition imposed, results of numerous pharmacological research has suggested potential applications in the treatment of psychological trauma, depressive phenomena, and especially in combating drug addiction [28,36,37,39]. Ibogaine is particularly effective when detoxifying from opiates. At the same time, ibogaine exhibits a certain cardiotoxicity [41] which has led pharmaceutical chemists to develop new derivatives that act on the opiate pathway and are safe for the cardiovascular system [40].

**Figure 3:**
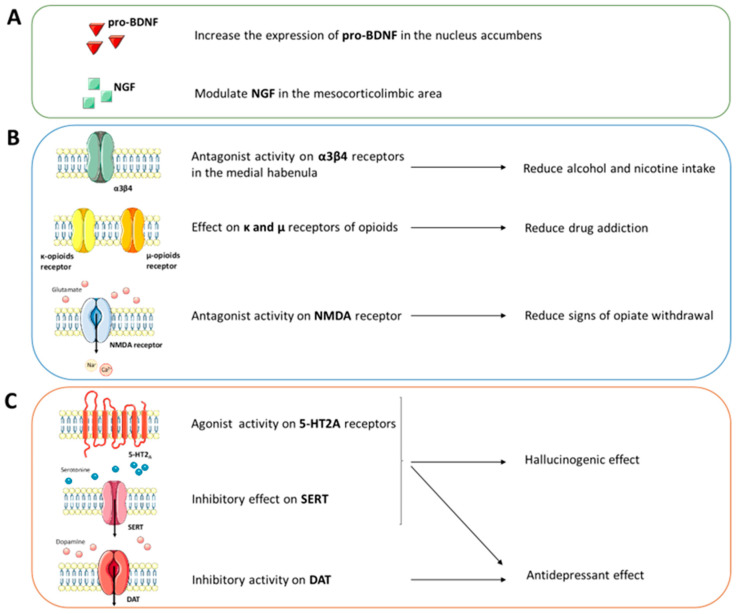
The receptor and molecular mechanisms involved in ibogaine activity requires: (**A**) neurotrophic factors, (**B**) opioid receptors and (**C**) transporters and receptors of monoamine. The figure was partly generated using Servier Medical Art, provided by Servier and licensed under a Creative Commons Attribution 3.0 unported license.

The molecular and receptor mechanisms involved in the action of ibogaine on addictions have yet to be well defined (Figure 3). However, some studies highlight the involvement of neurotrophic factors, monoamine transporters and receptors, and opioid receptors. For example, microinjection of ibogaine in the ventral tegmental area (VTA) induces a reduction of self-administration of ethanol in animal models [42,43]. This response is blocked after microinjection of anti-GDNF neutralizing antibodies into the VTA [44]. In addition, ibogaine has been shown to increase the expression of proBDNF in the nucleus accumbens, a brain area involved in neuronal reinforcement and gratification mechanisms [44]. These studies also report that neural growth factor (NGF) is modulated in the mesocorticolimbic area after treatment with ibogaine.

Reduced alcohol and nicotine intake has been associated with the antagonism of ibogaine derivatives to the α3β4 nicotinic acetylcholine receptors in the medial habenula [45,46,47,48]. Other studies have also reported the receptor affinity of ibogaine towards the κ and µ receptors of opioids involved in the reduction of drug addiction [47,48,49]. At the same time, ibogaine and its derivatives are considered N-methyl-D-aspartate (NMDA) receptor antagonists, similar to memantine, and are capable of reducing the signs of opiate withdrawal in mouse models [50,51,52,53].

As for the circuits involving monoamines, it seems that the agonism of ibogaine towards the signalling of serotonin (5-HT) is involved in the hallucinogenic and antidepressant effects. From one side, ibogaine acts as an agonist of the 5-HT2A receptors [54,55], on the other it shows an inhibitory effect on the SERT transporter, thus causing an increase in serotonergic tone [56,57,58]. The strong agonism of the 5-HT2A receptor is the basis of the hallucinogenic response induced by ibogaine. However, ibogaine and its derivatives do not show specific pharmacological profiles. Indeed, an inhibitory action was also highlighted on the monoamine transporters [59]. The high affinity of ibogaine and its derivatives to monoamine transporters could explain the observed antidepressant effects, as has also been elucidated by structural studies (see the *Structural and Computational Studies* section).

Pharmacokinetic and pharmacodynamic studies have focused on the effects of ibogaine and its derivatives on abstinence crises in humans [35,39,60]. In these studies, the subjects were exposed to oral administration at doses between 500 and 800 mg per individual (70 kg) [39,60]. The C_max_ for ibogaine and its derivatives ranged from 30 to 1250 ng/mL and a T_max_ approximately 2 h and 5 h after ingestion, respectively. Furthermore, the accumulation of ibogaine in adipose tissues suggests its slow release over time. This considerable inter-individual variability has complicated the interpretation of data on the efficacy of ibogaine and derivative treatments.

Despite the observed intra-individual variability, observational studies on the subjective effects of ibogaine are very consistent and have divided the experience after its administration into three phases [8,36,38]. In the first phase (up to 8 h after administration), the subject enters a dream state experiencing alterations in sensory perception and past memories of his/her life. The second phase (up to 20 h after administration) is emotionally neutral and reflective. In the last phase (up to 72 h after administration) the subject shows increased self-awareness and meaning in life, accompanied by greater excitement and disturbed sleep.

### 2.3. Structural and Computational Studies

The pharmacological action of ibogaine and its main circulating active metabolite, noribogaine, is very complex to understand because they interact with different receptors and transporters present in the CNS [44].

Even though action of ibogaine and noribogaine at various receptors and transporters has been known in the literature for a long time, in-silico investigations for the characterization and study of the interactions present in the ibogaine–target complexes have been developed in recent years in parallel with the evolution of computational techniques.

Given the involvement of ibogaine and its metabolites in mood disorders, the molecular docking approach has focused on the human serotonin transporter, hSERT. hSERT is a member of the neurotransmitter sodium symporters (NSSs) family, which is composed of twelve transmembrane secondary active transporters that utilize sodium (Na^+^) and chloride (Cl^−^) gradients to promote the transport of neurotransmitter across the membrane in the extracellular fluid [61,62] while e potassium ion (K^+^) antiport stimulates the transport process in the cytoplasmic environment [63,64]. hSERT is a major target of antidepressants and of ibogaine metabolites. The function of NSSs can be modulated by a set of small molecules, which control the availability of neurotransmitter at synapses. hSERT functioning can be summarized with the serotonin reuptake process [63]. Given the association between hSERT’s mechanism of action and different conformational states, Coleman et al. were able to resolve the structure of three of these hSERT-ibogaine arrangements, which were deposited in the Protein Data Bank (www.rcsb.org; accessed on 30 November 2022) and are identified by the following codes: 6DZY (outward-open), 6DZV (occluded), and 6DZZ (inward-open) [57,62,65]. Coleman and his group, based on in-silico and in-vitro studies, highlighted the way that ibogaine can be considered a non-competitive inhibitor that interacts at the level of the active site since it can bind to all three of these conformational rearrangements [66].

In this context, the results of the molecular docking study started with the identification of the hSERT’s binding site, which comprehends the following residues: Tyr95, Ala96, Asp98, Ile172, Ala173, Tyr175, Phe335, Ser336, Gly338, Phe341, Ser438, Gly442, Leu443, Thr497, Gly498 and Val501.

**Figure 4:**
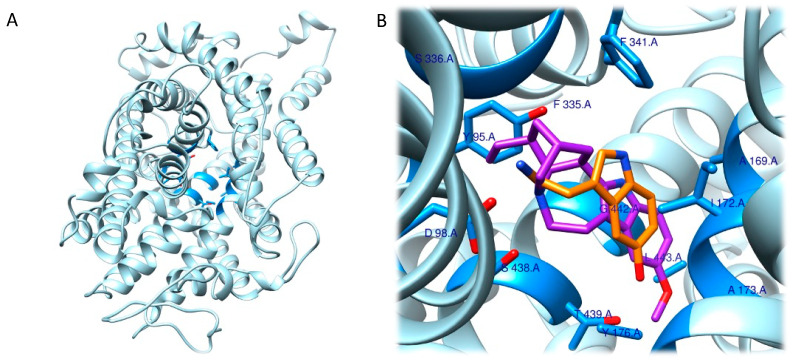
(**A**) Three-dimensional model representing hSERT (PDB ID: 6DZZ). Residues coloured in blue are those located in a zone within 5 Å of the serotonin. (**B**) Magnification depicting ibogaine (in purple) and serotonin (in orange) fitted into the central binding pocket of hSERT. The image was obtained by aligning the PDB ID 6DZZ and 7MGW structures. The highlighted residues are those most relevant to the interaction of the two molecules [57]. The superimposition (RMSD 1.152 Å) was completed using the MatchMaker tool in UCSF Chimera [70]. The artworks were produced using the same software.

Firstly, they found that the tricyclic ring system of ibogaine resides between the aromatic groups of Tyr95 and Tyr176 in the outward-open and occluded conformations. Further, when ibogaine docks in the binding pocket, it shows that there is a connection at the level of the tertiary amine of ibogaine and the Asp98 residue. Besides this, all three models of the SERT–ibogaine complex shared a common interaction pattern: Phe335 undergoing conformational changes from outward-open and occluded to inward-open, where it moves further into the binding site, ultimately blocking ibogaine release from the extracellular side while rearrangements of the inward-open conformation disrupt interactions of Tyr95 and Asp98 with ibogaine. Therefore, when there is a switch from an outward- to an inward-open conformation, ibogaine can change its orientation and switch toward to the cytoplasmic permeation state. Further, the methoxy-group of ibogaine, which protrudes into a cavity near to Asn177, Ile172, and the aromatic ring of Phe341, was preserved in all the assumed conformations (Figure 4A,B). The research then focused on the accessibility of ibogaine with respect to the position of the intracellular and of the extracellular gates present in the transporter [67].

The authors note that, in accordance with their previous work, when the ibogaine–hSERT complex is in the outward-open state, it assumes a conformation similar to that shown in the structure of paroxetine-bound hSERT [67]. From this evidence, they hypothesized that ibogaine can enter the binding site from the extracellular side because the gating residues Tyr176-Phe335 and Arg104-Glu493 lead to an open gate state, while the closed intracellular gate prevents exposure to the cytoplasm, leading to formation of the occluded conformation and a closure of the extracellular gate. All these events prevent ibogaine’s entry into the central binding site. Moreover, these data have been supported by molecular dynamics simulation experiments [57].

Importantly, the authors, using structural studies, were able to confirm the presence of an allosteric site in hSERT that is reported to modulate the association and dissociation processes of substrates in the central binding site and allow the definition of ibogaine as a non-competitive inhibitor [66,67,68,69]. According to this information, the authors understood that, when gating residues get closer, structural rearrangements result in the collapse of the allosteric site and, consequently, in a reduced solvent accessible surface area (SASA) for ibogaine access (from 1448 to 1247 Å^2^). Additionally, the conformational transition from occluded to inward-open conformations was investigated, and of note was a further reduction in the SASA value of the allosteric binding site (973 Å^2^) and in the distance between the gating residues Arg104-Glu493 and Tyr176-Phe335. All these events lead to an increased accessibility to the central binding pocket since they drive the opening of the cytoplasmic-permeation pathway.

Eventually, the authors speculated that, because it was impossible for ibogaine to access the central binding pocket of the occluded hSERT, it might bind to the inward- or outward-open conformation and remain bound there through the allosteric binding site so that it could then access the active site in a second step through conformational changes. Another aspect was then considered: the steric bulk of ibogaine (310.4 g/mol) is higher than that of serotonin (176.2 g/mol), which excludes it from being a competitive serotonin substrate candidate because it cannot fit completely into the central binding site. This further supports the hypothesis that considers ibogaine to be a non-competitive ligand for the central binding site capable of stabilizing the inward- and outward-open conformations of hSERT. The information obtained is crucial for future studies aimed at understanding the complete functioning of small molecules, such as ibogaine, that are capable of selectively binding the pharmacological target hSERT and for designing or optimizing ligands [57].

### 2.4. Therapeutic Hypotheses

The most studied therapeutic applications of ibogaine have focused on assessing its effects as a treatment for substance use disorders. Over the recent years, ibogaine has been taken into consideration as a therapeutic agent, able to treat or assist the treatments for drug addictions such as heroin [71], cocaine [72], methamphetamine [73], cannabis and crack [74]. The advantage of the use of ibogaine as a therapeutic for drug addiction resides in the fact that a single large dose seems effective in blocking withdrawal and reduces cravings in drug-dependent individuals [72], contrary to standard pharmacological treatments which typically require a prolonged administration. Indeed, ibogaine administration, unlike commonly used treatments such as methadone, shortens the time needed for withdrawal to 2–3 days, effectively speeding up the detoxification process [72].

Among the latest studies, that undertaken by Brown and Alper (2018) showed that a single dose of ibogaine (1540 ± 920 mg) reduced opioid withdrawal symptoms in 30 individuals with opioid use disorder (OUD), with subsequent detoxification effects sustained for 12 months post-treatment [28]. In line with these results, Noller et al. (2018) recently showed that a single ibogaine administration (200 mg capsules of ibogaine hydrochloride via oral administration) in 14 patients not only ameliorated withdrawal symptoms and led to sustained reduced opioid use over time, but also led to a collateral reduction in depression symptoms [75]. Another recent study [76] examined the opioid cravings and withdrawal symptoms in 50 patients with OUD undergoing detoxification treatment for one week with ibogaine (18–20 mg/kg of ibogaine hydrochloride via oral administration). At 48 h after the administration, withdrawal symptoms and cravings were significantly lower compared to the baseline, with the majority of patients exhibiting mild to nonclinical signs of cravings. Wilson et al. (2021) found similar results in case reports of two individuals with OUD [77]. In the first case, the patient was able to completely refrain from opioid use within 5–6 days of starting the ibogaine treatment, maintaining the abstinence for three years. In the second case, the patient took multiple doses of ibogaine over the course of four months, after which they stopped all non-medical opioids, and maintained abstinence for two years.

The largest study to date is an open label case series of patients (*n* = 191) diagnosed with opioid or cocaine dependence [72]. Individuals received, under medical assistance, gel capsules of ibogaine (8–12 mg/kg ibogaine hydrochloride), a dose range that was shown to be effective in blocking opioid withdrawal symptoms without side effects. The authors report that a single administration of ibogaine is effective for the detoxification process: subjects undergoing the treatment reported significantly decreased drug cravings and opioid withdrawal at 25–36 h after the last opioid dosage compared to baseline. They also reported a substantial decrease in scores of depression and anxiety and an improvement in overall mood one month after treatment.

In recent years, along with the growing use of psychedelics in the treatment of psychiatric disorders, research about the therapeutic effects of ibogaine has begun to take its first steps in this direction. For example, Fernandes-Nascimento et al. (2022) recently reported a single case study of ibogaine microdosing for the treatment of type II bipolar disorder (BPD) [78]. Microdosing, a growing trend in the psychedelics field, is defined as recurrent administration of small doses of psychedelic drugs, with little to no identifiable acute drug effects, with the intention of improving mental health and general wellbeing, enhance cognitive performances and boost mood [79,80]. The authors reported a significant improvement of symptoms in a 47-year-old woman with a 20-year history of BPD treated for 60 days, with two daily administrations of ibogaine (4 mg per administration, approximately 1% of a full conventional single dose). This finding is consistent with previous preclinical studies undertaken in rats [81], which characterized behavioural effects induced by acute ibogaine and noribogaine (20 and 40 mg/kg, i.p., single injections for each dose) in rats, assessing depression-like symptoms using the forced swim test. Both drugs induced a dose- and time-dependent antidepressant effect, without significant changes in locomotor activity.

Taken together, these studies demonstrate that the alkaloid ibogaine displays a potentially wide range of therapeutic effects, especially regarding its anti-addictive properties. Different open label studies have demonstrated its efficacy in ameliorating the symptoms of opioid withdrawal, cravings, and depression. In contrast to research in the realm of addiction, the anti-depression and anti-anxiety effects of ibogaine have not yet been investigated. Despite the lack of rigorous clinical trials, published research indicates that ibogaine could be an effective therapeutic agent, in particular regarding OUD, with sustained effects in the long run.

## 3. *Echinocactus williamsii*

### 3.1. Ethnobotany

**Figure 5:**
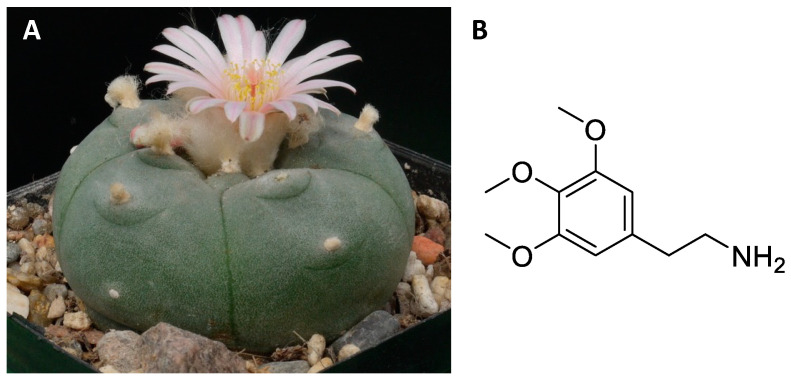
Flowered *Echinocactus williamsii* (**A**); chemical structure of mescaline (**B**).

The genus *Echinocactus* includes a dozen perennial cacti originating from Central America and South America with a globular or cylindrical structure, with slow growth, which can reach a diameter of 90 cm and develop a fleshy root [82,83,84]. Specifically, *Echinocactus williamsii* (Figure 5A), also called peyote, is a small cactus that grows in the Rio Grande valley and neighbouring deserts [82,83,84]. The psychoactive alkaloids are concentrated mainly in the epigeal part [85]. In particular, the “vegetable buttons” known as mescal correspond to the tips of the cactus cut and left to dry in the sun and subsequently used by shamans for mystical-religious rites [8,86,87,88,89]. Furthermore, in some Central American locations it is eaten fresh, dried in paste or as tea [8]. About 20 alkaloids are extracted from *Echinocactus williamsii*, the most important of which is mescaline [85] (Figure 5B). Mescaline is an alkaloid, biosynthetically derived from amino acids, with a chemical structure similar to monoamine neurotransmitters, and psychomimetic activity [85,90]. It is used by the natives of Central America both for its exciting and intoxicating properties and for the attractive hallucinations it causes [8,86,87]. In particular, it is capable of altering the basal psychic state leading to a condition of psychomotor arousal with euphoria and lively abnormal psychosensory manifestations (olfactory, auditory and above all visual hallucinations), symptoms of depersonalization and alterations in space-time orientation [90,91]. Some hallucinatory phenomena, called synesthetic phenomena, are characteristic, consisting in the fact that a perception, for example auditory, causes lively chromatic sensations. The substance is active at a dose between 100 and 400 mg per person orally [90,92].

### 3.2. Central Nervous System Pathways

Regarding the pharmacological aspects, mescaline is considered an agonist of the 5-HT2A and 5-HT2C receptors of serotonin, and of the α2A adrenergic receptor [90,93,94,95]. In contrast, mescaline shows low affinity for other serotonergic receptors and for dopamine receptors and monoamine transporters [85,91,93,94,95]. The hallucinogenic effects appear to be regulated by mescaline agonism towards the 5-HT2A receptor [85,95]. However, it has been observed in animal models that blockade of dopamine receptors with haloperidol can halt the characteristic effects of mescaline, suggesting the additional involvement of other neuronal pathways [91].

The half-life of mescaline varies in the animal models studied: in cats it is 2 h; in monkeys it can reach 18 h after intraperitoneal administration. The LD50 for mescaline also varies between species, being around 30 mg/kg in monkeys, 54 mg/kg in dogs, and 157 mg/kg in mice (all intravenously administered) [91]. Mescaline is rapidly absorbed from the gastrointestinal tract and distributed mainly to the liver and kidney in murine models [91]. About 40% of absorbed mescaline is excreted unchanged in the urine. The remaining mescaline binds to liver proteins which increase its half-life [91].

In rats, mescaline dosages of between 10 and 100 mg/kg have been reported to induce an anxiolytic action, motor hyperactivity, and a propensity for social interaction [91,93,96,97]. These effects reach their peak around 1 h, last for around 2 h and are dose dependent. Regarding hallucinogenic effects, all psychedelic 5-HT2A receptor agonists induce head-twitch behaviour in both mice and rats [90,97]. This behaviour is not observed with selective 5-HT2A receptor agonists. In rats, head-twitch behaviour was observed after the administration of 10, 50, and 100 mg/kg of mescaline, reaching the peak of the effect after 1 h [90,91]. In mice, mescaline induces the head-twitch behaviour at around 10 mg/kg with an inverted “U” shape curve that is dose dependent. This “U” shape response has also been observed in the locomotor behaviour of mice [97].

In humans, mescaline has a mean half-life of approximately 6 h, and its metabolites are excreted in the urine, plasma, and cerebrospinal fluid [91,98]. About 80% is found in human urine 24 h after oral administration of mescaline, and more than 90% within 48 h [91,96,98]. Sensory synaesthesia is among the effects reported by subjects receiving mescaline. Healthy individuals who listened to music after a mescaline administration had visual effects such as intense chromatic perceptions, and kaleidoscopic and geometrizing visions of objects [16,89,99,100]. At the same time, some individuals perceived flavours after seeing certain colours. Along with visual hallucinations, mescaline can alter the perception of time, space and personality. For example, after administration of mescaline (5–10 mg/kg), some subjects perceived time faster and others slower [85,90,92,94,95,99]. Other individuals reported feelings of euphoria, ecstasy, and general well-being.

### 3.3. Structural and Computational Studies

Navarro et al., after having generated and validated a 5-HT2A receptor model by homology modelling studies, performed in-silico experiments, including docking, to evaluate the interaction of mescaline with this target [101]. Molecular docking studies have been carried out in two different ways. Firstly, using a protocol that involved a rigid docking mode (RRA), which gives the ligand greater degrees of conformational freedom, something that the receptor is not granted. The second docking was carried out in flexible mode (FRA) where, in addition to the ligand, degrees of freedom are also granted in the 4 Å vicinity of the binding site of the receptor.

**Figure 6:**
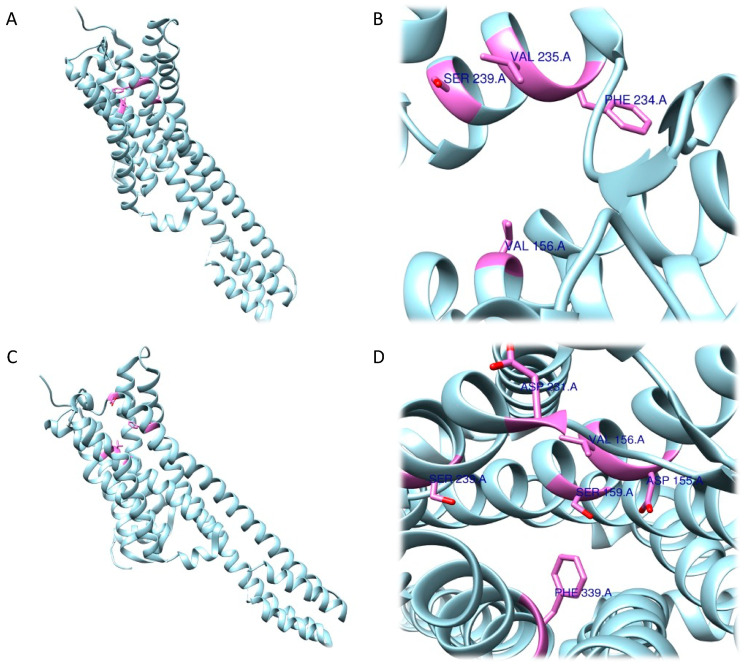
Three-dimensional model depicting the 5-HT2A receptor (PDB ID 7WC4). Residues coloured in pink are those involved in interactions with mescaline determined by the RRA method (**A**,**B**) and the FRA method (**C**,**D**) [70,101].

As expected, RRA and FRA methods displayed two different interaction patterns. Interactions obtained with RRA included one weak π–alkyl interaction between the aromatic ring of the ligand and Val156 (5.18 Å), three strong π–σ interactions between the aromatic ring and Val235 (2.77 Å), a hydrogen bond between one H atom from the NH_3_^+^ group and the backbone of Phe234 (2.26 Å), and another hydrogen bond between the O atom from the 5-OMe substituent and Ser239 (2.61 Å) (Figure 6A,B).

The FRA method provided different results in terms of interaction distances and involved residues. More specifically, one weak, four intermediate and one strong interaction were identified. The weak π–π interaction was identified between the aromatic ring and Phe339 (5.05 Å). The four intermediate interactions comprehend a π–π interaction between the aromatic ring and Val156 (4.83 Å), a H bond between the O atom (as an acceptor) from the 5-OMe substituent and the backbone of Ser239 (3.55 Å), a H bond between the 3-OMe substituent and Asp155 (3.34 Å), and a H bond between the 4-OMe substituent and Ser159 (3.50 Å). The strong interaction detected is a salt bridge interaction between one H atom from the NH_3_^+^ group and Asp231 (2.41 Å). Of note, the common residues involved according to both methods were only Val156 and Ser239 (Figure 6C,D).

### 3.4. Therapeutic Hypotheses

Mescaline, along with other psychedelics such as LSD and psilocybin, has been widely employed in psychiatric and therapeutic contexts, before its use was restricted by the Schedule I of the UN Convention on Drugs in 1967 [91]. Due to its regulation, clinical research over recent decades has been heavily limited [90]. Preclinical studies using animal models have been employed to investigate the effect of mescaline on behaviour. As mentioned previously, mescaline is able to induce an increase in locomotor activity and exploratory behaviour, although some studies report a dose-dependent reduction with an inverted U-shape curve (locomotion and exploratory behaviour increase at lower doses and decrease at higher doses) [102,103,104]. Mescaline is also able to induce an increase in animal reactivity [105].

Regarding mescaline’s therapeutic potentials, recent epidemiological studies support the anecdotal reports and preliminary research conducted in the 1900s. For instance, Agin-Liebes et al. (2021) conducted an epidemiological study with a sample of 452 adults, who completed an anonymous survey regarding the recreational use of mescaline in naturalistic settings [106]. The results indicate that mescaline administration is associated with improvements in general mental health and well-being, addressing issues such as anxiety, substance use disorder, depression, and PTSD symptoms. Most of the subjects with histories of these disorders (68–86%) reported improvement in their condition. Significant subjective improvements were reported in participants with histories of depression (*n* = 184), PTSD (*n* = 55), anxiety (*n* = 167), alcohol use disorder (*n* = 48) and drug use disorder (*n* = 58). The same dataset of 452 participants was used by Uthaug et al. (2022) for an epidemiological study, yielding similar results regarding the participant’s improvement in their psychiatric conditions [107]. The study also differentiated between mescaline from two different types of cactus species—*Lophophora williamsii* (Peyote) and *Trichocereus pachanoi* (San Pedro)—reporting no difference in drug effects.

The presented evidence—although of limited value—points to the notion that mescaline could have therapeutic properties worth investigating further. More research—especially controlled, randomized, double-blind clinical trials—is needed to determine its clinical efficacy and to determine where to place mescaline regarding its suitability for employment in therapies aimed at treating various psychiatric conditions.

## 4. Psychotria Viridis

### 4.1. Ethnobotany

**Figure 7:**
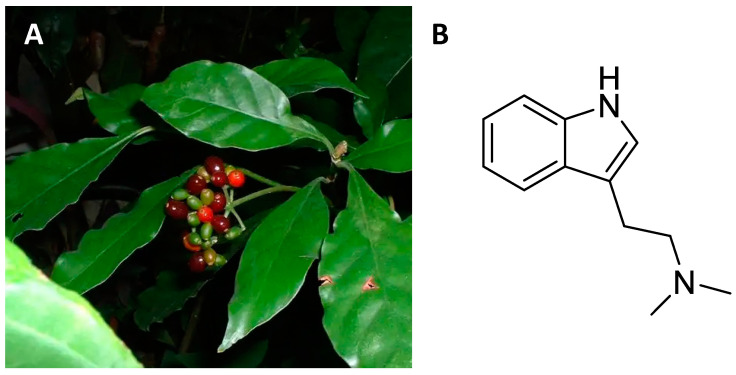
*Psychotria viridis* with red infructescences (**A**); chemical structure of N,N-dimethyltryptamine (**B**).

About 1400 shrubby species that grow in tropical areas around the world belong to the *Psychotria* genus [108,109,110,111,112,113,114,115]. In particular, *Psychotria viridis* (Figure 7A) is a member of the rubiaceae family that grows spontaneously in the humid areas of Central and South America [116]. The leaves of *Psychotria viridis* are rich in N,N-dimethyltryptamine (DMT, Figure 7B), a psychedelic alkaloid [117]. Depending on the different American ethnic groups, the dried leaves can be smoked or added to a drink that also contains *Banisteroriopsis caapi* leaves [118]. This drink is called *ayahuasca* and is used by shamans to contact deceased ancestors [119]. The hallucinogenic effect of ayahuasca is due to the presence of carbolines (harmine and harmaline) which inhibit the action of monoamine oxidase and enhance the action of DMT [9,10,118,119].

### 4.2. Central Nervous System Pathways

**Figure 8:**
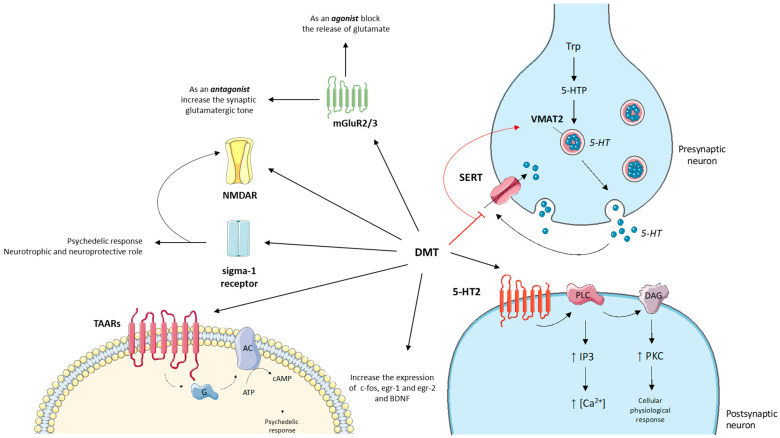
Graphical representation of DMT’s mechanism of action. DMT is a partial agonist of serotonin receptors (5-HT2) and its mechanism of action involves the second messenger pathway of PLC and A2 in post-synaptic neurons. DMT also acts as an inhibitor of SERT and VMAT2 transporters of serotonin at the pre-synaptic level. On the left of the figure, additional targets of DMT and their intracellular pathways are represented: mGluR2/3, NMDA, sigma-1 receptor and TAARs. Finally, DMT can promote synaptic plasticity by increasing the expression of the transcription factors c-fos, egr-1 and egr-2 and of the neurotrophic factor BDNF. The figure was partly generated using Servier Medical Art, provided by Servier and licensed under a Creative Commons Attribution 3.0 unported license.

Like most psychedelics, DMT is considered a partial agonist of serotonin receptors (Figure 8). Receptor binding studies have shown a DMT affinity to the 5-HT2A receptor of around 75 nM [117,120]. However, the 5-HT2A receptor appears to be necessary but not sufficient to explain the hallucinogenic phenomena reported in previous studies [121,122]. Indeed, the anxiolytic response observed in mouse models appears to be mediated by DMT binding to 5-HT1D and 5-HT3 receptors [117]. The psychedelic response to DMT can also be mediated by interaction with 5-HT1A and 5-HT2C receptors, although the head twitch response in mice is blocked only by 5-HT2A receptor antagonists [120,121].

The mechanism of action of DMT on 5-HT2 receptors involves the second messenger pathway of phospholipase C and A2. Phospholipases hydrolyse membrane lipids, generating inositol-1,4,5-triphosphate (IP3) and diacylglycerate, which leads to the activation of protein kinases and increase of intracellular calcium [117,122,123]. This mechanism of action is also common to other psychedelic molecules but needs further future studies.

DMT has also shown effects on the general serotonergic tone through the increase of serotonin at the synaptic level due to an inhibitory action on the SERT and VMAT2 transporters [124,125].

In the last 10 years some researchers have correlated the hallucinogenic effects after DMT administration to agonism towards glutamate receptors: mGluR2/3 and NMDA [125,126,127,128]. Metabotropic mGluR2/3 receptors are target sites for mediating hallucinogenic effects [125]. Agonists of the presynaptic mGluR2/3 receptors block the release of glutamate, while, on the contrary, the antagonists increase the synaptic glutamatergic tone, generating hallucinatory symptoms [125]. Depending on the dosage, DMT can show an agonist or antagonist profile at mGluR2/3 receptors, showing a heterogeneous behavioural phenomenology. However, heteroreceptor complexes consisting of the co-localization of mGluR2 receptors and 5-HT2A receptors can induce a second messenger cascade specific to the psychedelic phenomena associated with DMT administration [122,125,127].

DMT can also regulate the activity of ionotropic NMDA receptors directly, by modulating memory and learning processes, or indirectly, by activating the sigma-1 receptor [129,130,131,132]. The sigma-1 receptor is a chaperonin localized in the endoplasmic reticulum of cells of the cerebral or peripheral tissues [133]. Given the widespread distribution of the sigma-1 receptor, it has been studied in various diseases and neurobiological conditions such as addiction, depression, amnesia, pain, stroke and cancer [133]. DMT binds to the sigma-1 receptor at micromolar concentrations, contributing to the psychedelic response [130,132]. Agonism at the sigma-1 receptor is also involved in neurotrophic and neuroprotective processes [134,135]. Although totally convincing data on the direct involvement of DMT in the neuroprotective activity of the sigma-1 receptor has not been reported, it cannot be excluded [130,136].

Little research has been undertaken on the effects of DMT on acetylcholine signalling. The data collected show that DMT reduces the concentration of acetylcholine in the striatum but not in the cortex [125,127].

Around the 2000s, trace amine associated receptors (TAARs) were discovered such as those derived from the amino acid metabolism of phenylalanine, tyrosine and tryptophan (phenylethylamine, tyramine and tryptamine respectively) or derived from psychostimulants [137]. DMT can also bind to TAARs as an agonist causing activation of adenylate cyclase and subsequent accumulation of cAMP contributing to the psychedelic response along with 5-HT2A receptors [117,125].

Regarding the dopaminergic pathways, DMT does not bind to dopamine receptors and does not modulate its release at the synaptic level [125,138]. This point is fundamental, above all, for the aspects associated with the low desire to repeat the psychedelic experience after the first administration of DMT, contrary to what is observed with other psychotropic molecules.

Finally, DMT can promote synaptic plasticity by increasing the expression of the transcription factors c-fos, egr-1 and egr-2 and of the neurotrophic factor BDNF [117,139].

### 4.3. Structural and Computational Studies

Parts of the DMT structure are present in some important biomolecules, such as serotonin, making it a structural analogue that can interact with the same receptors located in the CNS as 5-HT1A, 5-HT1B, and 5-HT2A [140].

To understand the mechanisms and molecular interactions that occur between DMT and different macromolecular targets, computational studies have been carried out over the years. Navarro et al. (2015) attempted to understand the interactions between DMT and 5-HT2A receptor by means of RRA and FRA techniques [141].

**Figure 9:**
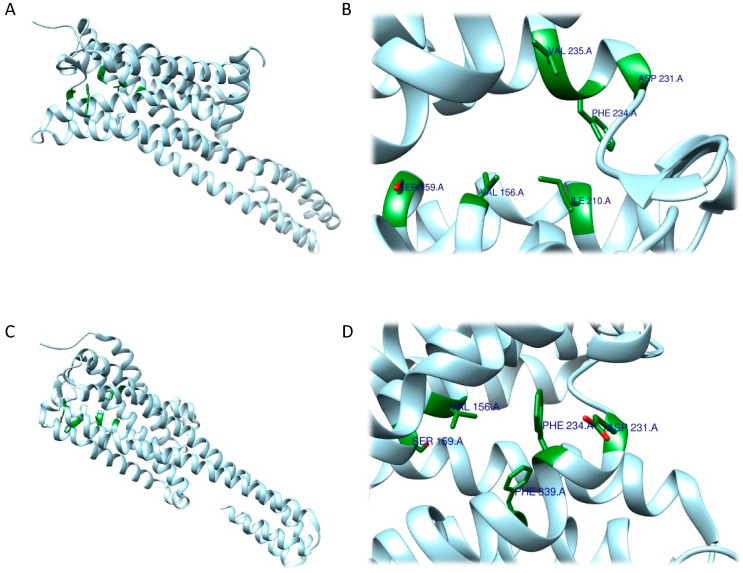
Three-dimensional model depicting 5-HT2A receptor (PDB ID 7WC4). Residues coloured in green are those involved in interactions with DMT determined by the RRA method (**A**,**B**) and FRA method (**C**,**D**) [70].

The RRA shows a π–σ interaction with Ile210 (2.83 Å), a H bond between the H(N) atom of the indole moiety and Asp231 (2.17 Å), a H bond between NHMe2 and Ser159 (3.43 Å), an amide–π stacking interaction with Phe234 (3.98 Å), an amide–π stacking interaction with Phe234 (4.12 Å), a π–alkyl interaction with Val235 (4.95 Å) and Val156 (5.01 Å), and a π–alkyl interaction with Ile210 (5.00 Å) and Val156 (4.95 Å). Once the interactions were identified, they were classified by the authors according to the distance between the atoms involved as strong (distance ≤ 3 Å), intermediate (distance between 3–5 Å), and weak (distance ≥ 5 Å). Overall, two strong, five intermediate, and two weak interactions were detected (Figure 9A,B).

FRA simulations showed a H bond of the H(N) from the indole moiety with Ser159 (2.3 Å), an attractive charge interaction of the N atom from NHMe2 with Asp231 (5.46 Å), a H bond between the H(N) from NHMe2 with an oxygen atom of Phe234 (2.90 Å), a π–alkyl interaction with Val156 (5.27 Å), a π–π interaction with Phe339 (5.32 Å) and another π–π interaction with Phe339 (4.84 Å). FRA results also show the presence of additional interactions between Asp231 and Phe234 and the NHMe2 group of the DMT molecule (Figure 9C,D).

Other information concerning the interactions of DMT, and other targets can be retrieved from the work of Contreras et al. who evaluated the DMT-5-HT1B complex through computational techniques such as docking and molecular dynamics [142].

**Figure 10:**
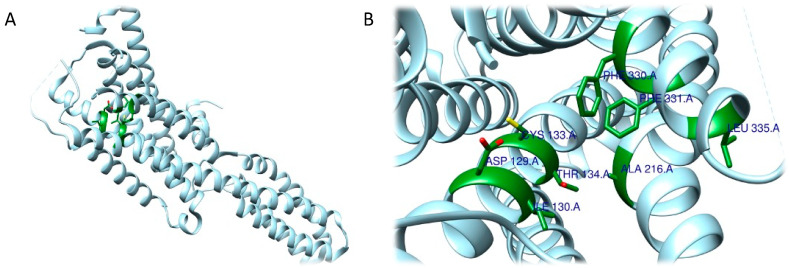
Three-dimensional model depicting 5-HT1B receptor (PDB ID 4IAR). Residues coloured in green are those involved in interactions with DMT, determined by Contreras et al. 2022 [70,142].

At a molecular level, the mechanism of the interaction between DMT and 5-HT1B is not yet fully known. In this regard, the aim of the work was to understand how the presence of DMT could somehow influence the stability of the receptor at the atomic level. To overcome the limitation of the current treatments there is an ongoing search for molecules that can contribute to the treatment of disorders such as anxiety and depression [143]. The resolved structure of the receptor (PDB ID: 4IAR) was used for molecular docking studies. The binding energy obtained from the docking was −6.65 ± 0.07 kcal/mol, comparable to that computed for serotonin (−6.50 ± 0.14 kcal/mol), a result that indicates a good value of binding energy. By comparing the behaviour of DMT and serotonin, the authors also confirmed that the two molecules share most interactions with the 5-HT1B receptor (Figure 10A,B).

Like serotonin, DMT interacts with Asp129 in 5-HT1B through a hydrogen bond, and Ile130, Thr134 and Thr355 residues are also involved in the interaction. As for non-hydrogen bonds, serotonin and DMT share π–sulphur interactions with Cys133, and aromatic interactions with Phe331. However, DMT also forms π–alkyl bonds with Ala216 and Phe330.

Additionally, molecular dynamics simulations within a timeframe of 100 ns allowed the assessment of the behaviour of the DMT-5-HT1B complex, which confirmed the stability of the assembly. Subsequently, the structure fluctuation was also evaluated via root mean square fluctuation (RMSF), which for DMT is between 4 and 5 Å. SASA was also evaluated and, again, was comparable to that of serotonin (22,000 Å^2^) throughout the simulation.

The total number of hydrogen bonds established during the molecular dynamic simulation of the DMT-5-HT1B complex was also considered and the results are similar to those of serotonin [142].

In previous studies concerning the 5-HT1B receptor, relevant residues for the interaction with the macromolecule itself were identified. These are residues Asp129, Ile130, Tyr208, Cys133, Thr209, Ser212, Trp327, Ala216, Phe331, Phe330, Asp352, Ser334, Tyr390, and Thr355 [140,144]. In this regard, it is also interesting to see how DMT can form interactions with some of the abovementioned residues. This helps to explain the high degree of stability that DMT has within the receptor. Subsequently, the fact that the SASA value is high agrees with the fact that the complex, in some areas, has a higher fluctuation value (represented by the RMSF parameter) at the important amino acids for the interaction.

The combination of these data with the results previously published in the literature, allowed the authors to understand that, since the number of hydrogen bonds is quite low, they do not substantially affect the binding mechanism, but they do contribute to conformational changes. Of note, further studies are surely needed to clarify the molecular mechanism of this relevant class of ligands on the 5-HT1B receptor.

### 4.4. Therapeutic Hypotheses

Although DMT is known for its psychedelic properties, latest research has shown its clinical utility, addressing a variety of medical conditions (especially those of the central nervous system). As a matter of fact, in the last decade DMT has been investigated for its neuroprotective effects, which are thought to be mediated by binding with the sigma-1 receptor [145]. In-vitro experiments have displayed DMT’s cytoprotective effect against hypoxia: diverse cell types (human cortical neurons derived from iPSC, dendritic cells, monocyte-derived macrophages) cultured in severe hypoxic conditions have been treated with DMT. Results suggest that DMT is able to prevent cellular stress and robustly boosts cell survival [146]. These data have been confirmed by in-vivo studies using animal models of cerebral ischemic injury. For instance, Szabò et al. (2021) have induced a global ischemic episode in rats under anaesthesia, coupled with the induction of spreading depolarizations (aiming increment metabolic stress and neurodegeneration), causing transient cerebral hypoxia [136]. Continuous administration of DMT via intravenous route (1 mg/kg/h) has been proven to be effective in reducing the depolarization by activation of the sigma-1 receptor and has shown neuroprotective properties, rescuing hippocampal cells from ischemia-induced apoptosis. Similar results were achieved by Nardai et al. (2020), who induced a transient cerebral occlusion in mice before treating them with a single dose (1 mg/kg, i.p.) followed by a maintenance dose (2 mg/kg-bw-h, via osmotic pump) of DMT [147]. Treated rats displayed less ischemic injury volume compared to controls, with better overall recovery. Moreover, the authors reported lower apoptotic protease activating factor 1 and increased BDNF levels in DMT-treated animals, possibly indicating an interplay between anti-apoptotic and neurotrophic factors. In this regard, recent research has uncovered the potential role of DMT in stimulating neurogenesis, a multi-faceted process that leads to the formation of new neurons. DMT has been shown to promote the in-vitro proliferation of neural stem cells and to stimulate the differentiation of these cells into the three main neural subtypes (neurons, astrocytes and oligodendrocytes) [148]. DMT is also able to activate the subgranular zone of the dentate gyrus in-vivo, a part of the hippocampal formation in which most adult neurogenesis takes place. This neurogenic propriety seems to have a robust functional effect, since DMT-treated mice (2 mg/kg, i.p., for 21 days) performed better in memory and learning tasks, for which the hippocampus plays an essential role [148]. The increased neurogenesis and subsequent cognitive improvement provided by DMT treatment could have profound implications for neurodegenerative diseases such as Parkinson’s (PD) and Alzheimer’s (AD) and for medical conditions defined by significant loss of neural cells in affected areas of the CNS, such as stroke. Previous literature [149,150,151,152] indicates that neurogenesis and neuroprotective factors could be implemented as a valid tool in the therapeutic strategies for psychiatric and neurological disorders, aiding in the restoration and preservation of residual functionality in the cerebral regions affected by the disease. This concept has recently been assessed using DMT-containing Ayahuasca in an in-vitro model of Parkinson’s disease. Katchborian-Neto et al. (2020) tested the neuroprotective effect of ayahuasca and its matrix plants, *B. caapi* and *P. viridis*, in a human neuroblastoma cell line (SH-SY5Y) with neurodegeneration induced by 6-hydroxyldopamine (6-OHDA), a well-known in-vitro PD model [153]. The lower doses of the compounds were effective in stimulating neuronal proliferation and displayed significant neuroprotective properties, assessed by improved cell viability and protection against 6-OHDA-induced cell damage. In the context of neurodegenerative disorders such as AD, PD and amyotrophic lateral sclerosis, recent research (for reviews, see [134,135]) suggests that acting on the sigma-1 receptor, for which DMT is an agonist, could be an effective therapeutic strategy, given the incredible versatility of this receptor and its role in mediating neuroprotection and maintaining neuronal homeostasis. In order to address these promising considerations, further work is essential to better uncover the potential role of DMT in the treatment of neurodegenerative disorders.

Along with clinical conditions of the CNS, researchers and clinicians have focused their consideration on the effectiveness of this substance for the treatment of psychiatric conditions and mental illnesses. DMT and, in particular, the DMT-containing Ayahuasca, have gained attention for their mood-stabilizing and relaxing proprieties, as reported by previous scientific research [154,155,156,157]. Palhano-Fontes et al. (2019) conducted the first randomized, placebo-controlled trial, which investigated the effects of a single dose of Ayahuasca (1 mL/kg, containing 0.35 mg/kg of DMT) in 29 patients affected by treatment-resistant depression [158]. The authors reported evidence of a robust, fast-acting antidepressant effect of the preparation, sustained throughout the period of observation (seven days) compared with placebo. These results have been confirmed by a subsequent study [159] in which patients with major depressive disorder (*n* = 7) were treated with an escalating dose of DMT (a first 0.1 mg/kg dose, followed by a 0.3 mg/kg dose, intravenous), distributed in two session 48 h apart. The authors reported a significant reduction in depressive symptomatology only one day after receiving the second DMT dose, corroborating the findings of Palhano-Fontest et al. about the rapid mood-stabilizing effects of the compound. In addition to these results, Almeida et al. (2019) tried to uncover the role between the depression-mitigating effects of ayahuasca and serum BDNF levels [160], given this neurotrophic factor’s potential role as a biomarker in depression: serum BDNF levels in patients with depression are lower than average and increase after treatment with serotonergic antidepressant [161,162]. Forty-eight hours after treatment (1 mL/kg of ayahuasca) BDNF serum levels were higher in both patients and healthy controls, compared to placebo, and just the experimental group (not the placebo group) displayed a significative inverted correlation between depression symptomatology and levels of BDNF.

Apart from clinical and preclinical studies, ayahuasca’s therapeutic potential seems to be confirmed by several observational studies, conducted on participants taking ayahuasca in naturalistic setting [163,164,165,166]. Although the clear limitation of observational studies, it is important to notice that this field of literature is unanimous in confirming the potential healing properties of ayahuasca and DMT, reporting robust improvements in psychopathological symptoms, boost in overall mood and improvements in mental health well-being that are persistent over time after a single dose.

All these results seem favourable in support of the use of psychedelics as antidepressant drugs, despite further research being necessary to corroborate previous findings and, most importantly, to determine whether the observed anti-depressant effects are stable over a longer period of time. Another aspect that should not be underestimated is the safety of treatments based on ayahuasca and DMT. Few clinical studies have emphasized the toxicological aspects of the use of ayahuasca and DMT. For this reason, particular attention must be given to the treatment with ayahuasca and DMT and the presence of a psychotherapist or psychiatrist is essential.

Additionally, supplementary investigations are required to establish whether more robust and effective outcomes can be achieved by a subchronic administration, compared to the single-dose administration employed in the previous studies. In this regard, pre-clinical studies conducted on the chronic administration of DMT seem promising in terms of ameliorating symptomatic conditions such as depression and anxiety. For instance, Cameron et al. (2019) tested the microdosing of DMT in adult rats, intraperitoneally injecting low doses of the substance (1 mg/kg) periodically (one dose every three days) for an extended period of time (two months) [167]. They found that the use of a sub-hallucinogenic dose in a chronic manner is efficacious in eliciting an antidepressant effect, without having impact on working memory and social interactions.

Apart from mood disorders, there has been speculation about a possible link between DMT and other medical conditions such as autism spectrum disorders (ASD). For example, Shomrat and Nesher (2019) propose a new view in this field, suggesting that DMT metabolism could be a factor in the structural alteration observed in autistic individuals [168]. Given that abnormalities in dendritic spine formation and cortical overgrowth are hallmarks of ASD [169] and that research evidence suggests that DMT has neuritogenic properties [170], the authors suggest that dysfunction of the pineal gland, the source of endogenous DMT, could potentially be involved in the etiopathogenesis of ASD.

Although promising, the latest research concerning the use of DMT as a therapeutic is still limited by the lack of placebo-controlled trials and research targeting various psychiatric conditions. Further investigations are required to unlock the full potential of this psychedelic in the clinical field.

## 5. Psilocybe Cubensis

### 5.1. Ethnobotany

**Figure 11:**
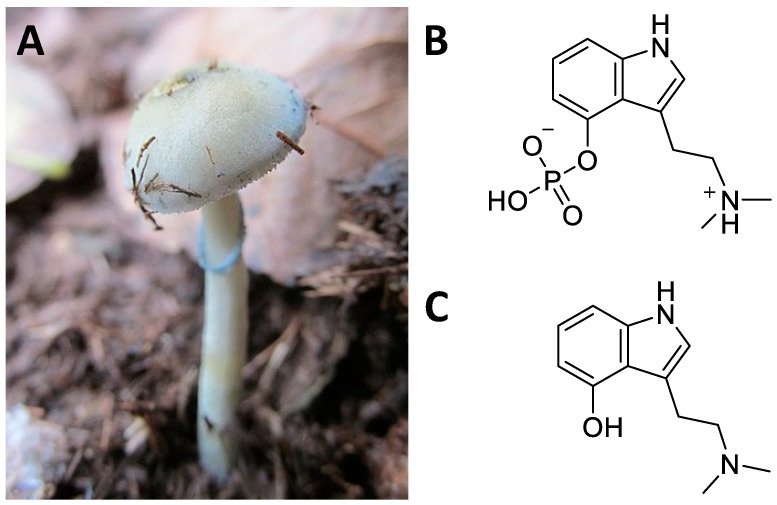
*Psilocybe cubensis* (**A**); chemical structure of psilocybin (**B**); psilocin (**C**).

Psilocybe mushrooms are Basidiomycota members of the family Strophariaceae [171]. The species of this genus are cosmopolitan, the best known are the *P. cubensis* (Figure 11A) and the *P. Mexicana,* which grow in Central America and have been historically used for shamanic rites [172]. Mushrooms are prepared in various ways depending on the shaman’s preferences [86]. They can be eaten fresh, dried, or infused and trigger hallucinogenic responses in users. The ritualistic use of Psilocybe mushrooms in Mesoamerica is documented by the 14th century Codex “Yuta Tnoho” or “Vindobonensis Mexicanus I”, which depicts a sacred ceremony where deities consume sacred mushrooms prior to the first dawn [173]. However, the discoveries of the Tassili mural in Algeria, reporting fungi associated with *P. mairei*, and the Selva Pascuala mural in Spain, a rock painting representing fungoid figures that have been associated with *P. hispanica*, date the use of these natural products back 7000–9000 years [174].

The ingestion of Psilocybe mushrooms induces hallucinations and synaesthesia resulting in a trance-like experience that is thought to allow dissociation of the soul from the body [173]. Traditionally, shamans have used these natural products as sacraments to enhance the healer’s divinatory capacities for different purposes, such as bodily ailment diagnosis and healing [175,176]. Apart from the ritualistic ones, other uses of Psilocybe mushrooms in the traditional medicine of Mesoamerica comprise the treatment of rheumatism, toothache and stomach pain, for example [173].

The psychotropic effects of Psilocybe mushrooms are attributable to two indole alkaloids known as psilocybin and psilocin (Figure 11B,C) [177]. Psilocybin is the phosphoric ester of psilocin which is present only in trace amounts. These compounds were first identified by Hoffmann and colleagues working on a sample of *P. mexicana* collected by Heim [178]. Together with the genus *Psilocybe*, mushrooms also belonging to the genera *Conocybe* and *Stropharia* show marked hallucinogenic actions [171]. *Conocybe* is known to contain psilocybin [179], and its analogue baeocystin [180]. On the other hand, thanks to the phylogenetic analyses based upon DNA sequence comparison, hallucinogenic *Stropharia* species have been reclassified into the genus Psilocybe [181].

### 5.2. Central Nervous System Pathways

After oral administration of increasing doses of psilocybin, it loses its phosphate group and is totally converted to psilocin in the acidic environment of the stomach or by alkaline phosphatase in the intestine and kidneys [182,183,184]. Therefore, evaluations of the pharmacological profile of psilocybin have been performed with its main derivative [182]. Psilocin is identified in plasma within half an hour of administration and reaches peak concentrations within three hours [184]. Plasma AUC increases in proportion to dose, indicating linear pharmacokinetics in doses between 0.3 and 0.6 mg/kg of psilocybin [183,184]. The average bioavailability of psilocin is around 50% and its average half-life is around three hours. More than 80% of psilocin is metabolized by glucuronidation and released in the urine as psilocin-O-glucuronide [183,184].

**Figure 12:**
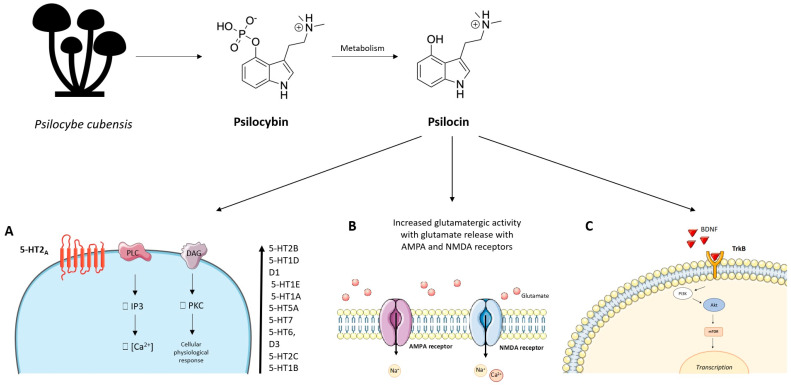
After oral administration, psilocybin loses its phosphate group and is totally converted to psilocin, which consequently represents the main derivative responsible for its pharmacological activity. (**A**) Psilocin has a good affinity for the 5-HT2A receptor, and this binding is responsible for the “mystical” hallucinatory effects induced by psilocin. In increasing order of affinity, psilocin can also bind to 5-HT2B, 5-HT1D, dopamine D1, 5-HT1E, 5-HT1A, 5-HT5A, 5-HT7, 5-HT6, D3, 5-HT2C and 5-HT1B receptors. (**B**) Activation of the 5-HT2A receptor in the prefrontal cortex by psilocin results in increased glutamatergic activity with glutamate release with AMPA and NMDA receptors on cortical pyramidal neurons. (**C**) Psilocin has been observed to exert its pharmacological action by enhancing neuroplasticity and neuritogenesis by acting through BDNF and mTOR pathways. This figure was partially generated using Servier Medical Art, provided by Servier and licensed under a Creative Commons Attribution 3.0 unported license.

Psilocin has also shown a good receptor affinity for the 5-HT2A receptor with the EC50 for receptor occupancy around 2 μg/L [185,186,187]. However, psilocin is considered a partial agonist of the 5-HT2A receptor (Figure 12). Indeed, compared with serotonin, psilocin shows an efficiency of less than 40% in the Ca^2+^ mobilization assay [188]. The link with the 5-HT2A receptor is responsible for the “mystical” hallucinatory effects induced by psilocin [185]. However, a certain psilocin receptor non-specificity common to many psychedelics has been confirmed. In increasing order of affinity, psilocin can also bind to 5-HT2B, 5-HT1D, dopamine D1, 5-HT1E, 5-HT1A, 5-HT5A, 5-HT7, 5-HT6, D3, 5-HT2C and 5-HT1B receptors [185,188].

The nonspecific binding of psilocin to different receptors can modulate the interactions between multiple neuronal pathways by altering the functional connectivity between brain areas [32,99,177,186,187,189,190]. These psilocin-induced alterations could also underlie its hallucinatory response. Some authors have observed an increase in glucose consumption in the prefrontal cortex, anterior cingulate, temporal cortex, and putamen after psilocybin administration [8,86,186,189,190]. The increase in glucose metabolic rate in these brain areas correlates positively with the “ego dissolution” due to the psychedelic response [189,190].

Simultaneously with the psychedelic effects, psilocin has shown antidepressant and anxiolytic effects at the basis of its therapeutic use. Indeed, psilocin, acting on serotonergic receptors, deactivates or normalizes the hyperactivity of the medial prefrontal cortex which is typically hyperactive during depressive phenomena [32,189]. This antidepressant action also seems to involve limbic areas including the amygdala, which is considered the centre of perception and processing of emotions. Psilocin causes an indirect variation of the dopaminergic and serotonergic tone in a differentiated way in some mesolimbic areas of the brain [191]. Indeed, after psilocybin administration, serotonin increases in the medial prefrontal cortex but not in the nucleus accumbens, while dopamine increases in the accumbens but not in the cortex [191,192]. This different response is related to the activation of 5-HT2A and 5-HT1A receptors in the different mesolimbic areas [185]. The increase in dopamine and serotonin is responsible for the increase in mood and psycostimulation. Furthermore, activation of the 5-HT2A receptor in the prefrontal cortex by psilocin results in increased glutamatergic activity with glutamate release with AMPA and NMDA receptors on cortical pyramidal neurons [192].

Psilocin has been observed to exert its pharmacological action by enhancing neuroplasticity and neuritogenesis by acting through the tropomyosin kinase B (TrkB) receptor and through the mammalian target rapamycin receptor (mTOR) [193]. Furthermore, psilocin can also increase the expression of neurotrophic factors such as BDNF, resulting in the increase of hippocampal neurogenesis and, at the behavioural level, the extinction of behaviours related to conditioned fear [193]. These effects on neurogenesis and neuroplasticity may underlie the potential therapeutic effects of psilocin in depressive and anxious states.

### 5.3. Structural and Computational Studies

Many computational studies have been performed to evaluate the behaviour of psilocin on serotonin receptors and to pave the way for new therapeutic approaches.

**Figure 13:**
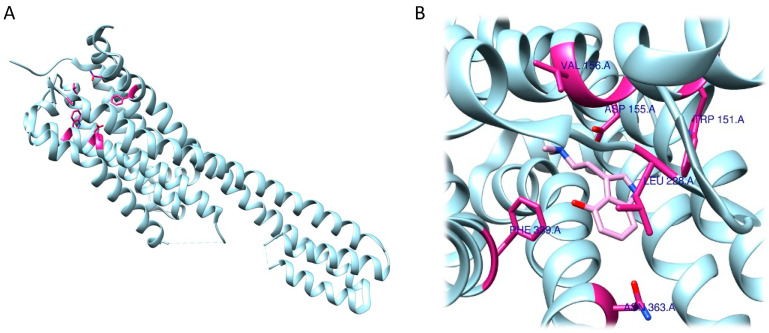
Three-dimensional model depicting the 5-HT2A receptor (PDB ID 7WC5) (**A**). (**B**) Detailed view of residues (in magenta) involved in interactions with psilocin (pink) determined by Cao et al. 2022 [70].

Concerning the 5-HT2A receptor, Cao et al. (2022) resolved and deposited the structure of psilocin-5-HT2A complex (PDB ID: 7WC5) [194,195]. The work performed by Cao et al. showed that, in addition to the central binding pocket, psilocin is also able to interact with a second binding site where the indole group fits into a pocket, described as an extended binding site, mainly containing hydrophobic residues. A salt bridge is formed between the basic nitrogen and the Asp155 residue and a hydrogen bond between Asn352 and the OH- group in the indole of psilocin was detected. Subsequently, during the analysis of the interactions between the 5-HT2A receptor and psilocin, additional residues involved in the formation of the complex, such as Val156, Phe339, Asn363, Leu228, and Trp151, were identified (Figure 13A,B) [195].

**Figure 14:**
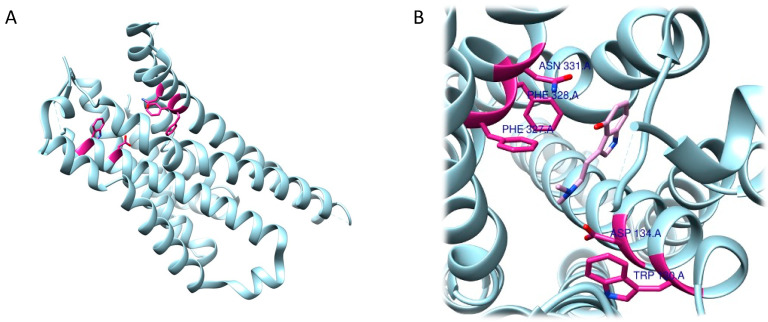
Three-dimensional model depicting 5-HT2C receptor (PDB ID 8DPG) (**A**). (**B**) Detailed view of residues (in magenta) involved in interactions with psilocin (pink) determined by Gumpper et al. 2022 [70].

The study of the role of psilocybin and of its active metabolite then continued, though focused on another receptor, 5-HT2C. In this case, molecular docking was enrolled, and Gumpper et al. (2022) showed that psilocin is also able to bind to the binding pocket of the receptor [196]. Here the compound establishes bonds with a salt bridge with Asp134 through the amine compound, a π–stacking interaction between the tryptamine ring and Phe328, and another bond between the indole -OH and Asn331. Further, other interactions with Trp130 and Phe327 were identified in the assembly (Figure 14A,B).

In order to validate the interactions that were found, the authors performed a molecular docking experiment in which they calculated a binding energy of −5.76 kcal/mol (Glide Docking Score) and an RMSD of 1.05 Å [196].

Also in this case, Gumpper et al. deposited the structure consisting of 5-HT2C and psilocin in the Protein Data Bank (PDB ID: 8DPG).

### 5.4. Therapeutic Hypotheses

Psilocybin is a medium-lasting, well-tolerated classic psychedelic that is potentially safe and effective [189,197,198]. Among psychedelics, psilocybin represents the path-opening drug in modern psychedelic-supervised therapy. In 2021, the FDA twice designated psilocybin the designation of “breakthrough therapy”, to accelerate its drug development and review process. At first, the FDA supported psilocybin for the treatment of depression and severe treatment-resistant depression. Subsequently, the FDA supported the Compass Pathways company in testing psilocybin as a promising therapeutic option for major depressive disorder (MDD). After the conclusion in 2022 of this clinical trial, in which 233 participants were enrolled, the authors were able to demonstrate that a single administration, though of a high dosage of psilocybin (25 mg), was able to reduce the Montgomery–Åsberg Depression Rating Scale (MADRS), a clinical parameter used to establish the depression severity, by 12 points [199]. Another important finding, which emerged from this study but that needs to be further researched, is the high incidence rate (77%) of adverse events, including headache, nausea, dizziness, suicidal ideation, and self-injury, which occurred in all dose groups. Unfortunately, studies on the safety of psilocybin treatments have only been performed in healthy individuals. At the same time, clinical studies on the efficacy of psilocybin have not yet evaluated the parameters of safety.

Although the safety and efficacy of psilocybin as an antidepressant are the focus of most recent clinical trials (more than 100 clinical trials have been registered), there are still no definitive data that can establish whether psilocybin can offer substantial clinical improvement over existing antidepressant therapies [200,201]. It has recently been reported that the antidepressant response of psilocybin is not statistically stronger than the conventional antidepressant escitalopram [202].

Across two different clinical trials, post-treatment fMRI data confirmed that psilocybin can modify and increase the brain network organization in patients treated with 2 × 25 mg oral psilocybin, three weeks apart, as well as six weeks of daily placebo.

To further understand whether psilocybin exerts its antidepressant effect by increasing synaptogenesis, in an upcoming, open-label study (ClinicalTrials.gov Identifier: NCT05601648 November 2022) participants will undergo positron emission tomography (PET) imaging before and one week after 25 mg psilocybin treatment by using 11C-UCB-J, a radiotracer that binds to SV2A, which is itself a marker of synaptic density and synaptogenesis. This study will allow researchers of the Washington University School of Medicine to assess the relationship between neurotrophic and antidepressant effects produced by psilocybin.

In addition, the promising neurotrophic and anti-inflammatory properties of psilocybin are generating interest as a therapeutic hypothesis for neurodegenerative diseases, although preclinical results are consistent, clinical trials are nowadays limited and mostly relate to the treatment of depression associated with neurodegenerative diseases such as Parkinson’s and Alzheimer’s [31,203]. Clinical evidence has supported additional therapeutic opportunities for this “magic mushroom” drug [204,205].

Again, the FDA supported an investigational new drug (IND) clinical trial to explore how psilocybin-assisted therapy impacts the treatment of anorexia nervosa (ClinicalTrials.gov Identifier: NCT04505189). For this study, a small group of patients with a primary diagnosis of anorexia nervosa as defined by DSM-V criteria will take part in eight study visits, including three psilocybin dosing sessions with varying doses up to the maximum of 25 mg per single session.

Studies are underway that aim to assess whether psilocybin is more feasible, tolerable, and efficacious for the treatment of post-traumatic stress disorders when administered alone or in combination with assisted therapy.

Concerning alcohol, smoke and substance abuse and anxiolytic effects, psilocybin effects are in line with the results demonstrated by other psychedelic drugs, as the long-lasting improvements were detectable up to six months after psilocybin administration [206,207,208]. Although several challenges are already present in effectively transposing psilocybin into the clinic, great efforts in clinical trials have been made to define what the optimal psilocybin formulation might be. This is the proper goal of a small interventional clinical trial planned in 2022 where safety, adverse effects, and physiological and psychological effects of PEX20 (Oral Psilocin), PEX30 (Sublingual Psilocin), and PEX10 (Oral Psilocybin) will be compared (ClinicalTrials.gov Identifier: NCT05317689).

Therefore, despite the lack of double-blind randomized studies, several clinical trials recently completed or underway shared the common goal to increase the knowledge of this highly attractive molecule with high therapeutic potential and for which several states are questioning its “decriminalization.”

## 6. Claviceps Purpurea

### 6.1. Ethnobotany

**Figure 15:**
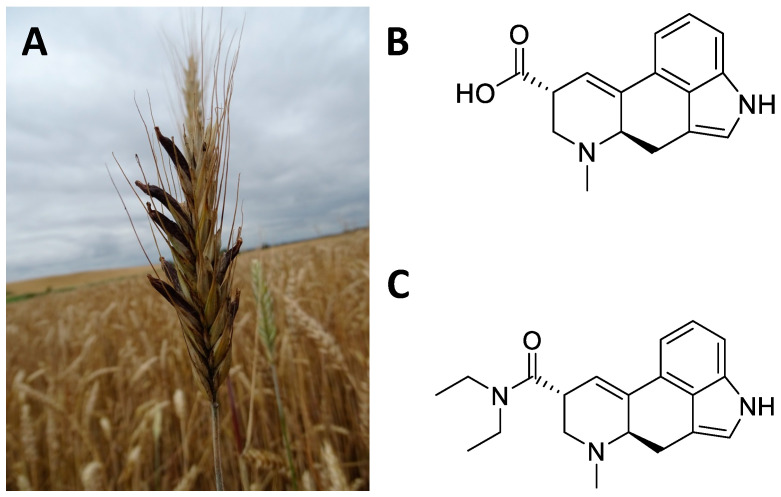
Sclerotium of *Claviceps purpurea* on an ear of grass (**A**); chemical structure of lysergic acid (**B**); lysergic acid diethylamide (synthetic compound) (**C**).

*Claviceps purpurea* is a fungus, belonging to the Ascomycetes Clavicipetali, which infests cereal crops and in particular rye [209] (Figure 15A). The sclerotium, *Secale cornutum*, is the richest part of the alkaloids (Figure 15B). These include ergolinic alkaloids derived from lysergic acid such as ergotamine, ergometrine, ergocristine, ergocriptine, and ergoconine [210]. Alkaloids have found multiple applications in the cardiovascular and gynaecological fields [211]. However, the greatest impact on the CNS came with the semi-synthetic synthesis of D-lysergic acid diethylamide (LSD, Figure 15C), deriving from lysergic acid. Indeed, in 1938 the chemist Albert Hofmann synthesized LSD for the first time in the Swiss laboratories of the Sandoz AG Pharmaceutical Company, deriving from lysergic acid, and accidentally tested its hallucinatory and psychedelic effects [212,213]. LSD has had a major social impact since the 1960s by profoundly influencing Western culture [212,213]. The cerebral effects of LSD concern the emotional–ideational aspects and above all sensory perception (colours are perceived more vividly) [214,215,216,217]. These hallucinogenic effects affect the sight, hearing, touch, and perception of one’s body. Furthermore, subjects who used LSD experienced introspective trips that enabled them to perceive inner problems and reality from other points of view, beyond the usual schemes [214,215,216,217].

### 6.2. Central Nervous System Pathways

LSD is totally absorbed in the intestine after oral administration. Absorption can be affected by the pH of the stomach and duodenum [218,219]. Indeed, the administration of LSD with food induces plasma concentrations that are halved when compared to administration on an empty stomach [220]. The different routes of administration do not determine qualitative differences in the hallucinatory effects of LSD but only in the intensity and speed of onset [220,221,222]. LSD can cross the blood–brain barrier as previously observed in mice, rats, cats and monkeys [220,221,223]. In humans, after an administration of 2 μg/kg i.v. LSD levels were approximately 7 ng/mL after 30 min and disappeared after 10 h [220]. Some variation was observed between species in the half-life of LSD at the same dosage and route of administration: 7 min in mice, 130 min in cats, 100 min in monkeys, and 175 min in humans [223]. LSD is almost completely metabolized to 13- and 14-hydroxy-LSD and their conjugates glucuronic acid, 2-oxo-LSD, and nor-LSD and only a small part of the unchanged drug is excreted [224]. LSD can be metabolized in humans to 2-oxo-LSD and 2-oxo-3-hydroxy-LSD by certain NADH-dependent liver microsomal enzymes [220,221,223]. In addition, lysergic acid ethylamide (originating from dealkylation of the diethylamide radical at position 8 of the side chain), nor-LSD and di-hydroxy-LSD have also been identified in human blood and urine [218,219]. These metabolites, and LSD itself, can be identified in urine for up to four days after ingestion.

In general, LSD users have reported that the ingestion of about 75–150 μg of LSD profoundly alters their state of consciousness, leading to euphoria towards affective people, and greater introspective capacity [213,214]. Users show altered sense perception in their bodies, with hallucinations and synaesthesia lasting up to 10 h [92,215]. Some authors have reported traumatic experiences (“bad trips”) after the administration of LSD, but the treatment was not performed under controlled conditions or assisted by medical personnel [23,212,213,214,215]. For this reason, the context in which the administration of psychedelic substances takes place is essential for achieving the desired therapeutic goals.

**Figure 16:**
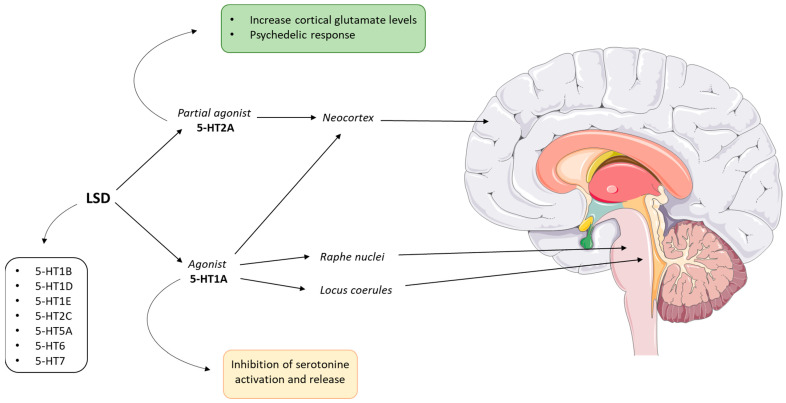
LSD can agonistically bind the serotonin 5-HT1A receptors in the locus coeruleus, raphe nuclei, and cortex causing the inhibition of serotonin’s activation and release. Simultaneously, through the thalamic afferents, LSD can activate the 5-HT2A receptor, inducing an increase in cortical glutamate levels. Furthermore, it has been observed that the activation of 5-HT2A receptors in the cortex triggers the psychedelic response in genetically modified mice expressing 5-HT2A receptors only at the cortical level. Moreover, LSD also has a high affinity for other serotonergic receptors such as 5-HT1B, 5-HT1D, 5-HT1E, 5-HT2C, 5-HT5A, 5-HT6 and 5-HT7. This figure was partially generated using Servier Medical Art, provided by Servier and licensed under a Creative Commons Attribution 3.0 unported license.

Serotonin signalling is also involved in the psychedelic response to LSD [95]. Serotonin is produced in the midbrain neurons of the raphe nuclei and is released from neuronal projections in the locus coeruleus, brainstem, and cortex. Neurons in the locus coeruleus control the release of norepinephrine, regulate the sympathetic nervous system, and extend into the cerebellum, thalamus, hypothalamus, cortex, and hippocampus [56,225]. LSD is a 5-HT1A receptor agonist in the locus coeruleus and raphe nuclei. LSD agonism in these areas interferes with serotonin signalling [226,227,228]. Furthermore, LSD is considered a partial agonist of 5-HT2A receptors, especially those expressed on neocortical pyramidal cells [227] (Figure 16). Through the thalamic afferents, LSD can activate the 5-HT2A receptor and induce an increase in cortical glutamate levels [229,230]. Glutamate release could be responsible for LSD-induced alteration of corticocortical and corticosubcortical transmission [231]. It has been reported that the difference between 5-HT2A receptor agonists with hallucinogenic activity and those without is due to the different activation of the heterotrimeric proteins G_i/o_ and G_a/11_ [232,233]. Furthermore, the activation of 5-HT2A receptors only in the cortex is sufficient to trigger the psychedelic response in genetically modified mice that express 5-HT2A receptors only at the cortical level [227]. This implies that cortical pathways are the medial site of the hallucinatory response following LSD administration. However, LSD is not a molecule that selectively binds to specific receptors and therefore the understanding of its mechanisms of action is still not entirely clear. Indeed, LSD also has a high affinity for other serotonergic receptors such as 5-HT1B, 5-HT1D, 5-HT1E, 5-HT2C, 5-HT5A, 5-HT6 and 5-HT7 [94,213,217,222,229,230,233].

### 6.3. Structural and Computational Studies

**Figure 17:**
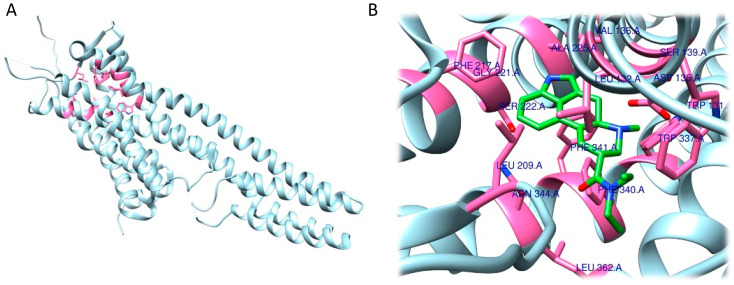
Three-dimensional model depicting 5-HT2B receptor (PDB ID 5TVN) (**A**). (**B**) Detailed view of residues (in pink) involved in interactions with LSD (green) determined by Wacker et al. 2017 [70].

To assess the action of the LSD, at the level of 5-HT2B receptor, Wacker et al. (2017) characterized the complex through computational studies and structural resolution techniques [234]. Their analysis revealed that LSD can bind to the receptor within the orthosteric binding site with a volume of 2898.7 Å^3^ [235]. The ligand has been shown to be able to insert itself into the orthosteric binding pocket through a salt bridge that occurs between Asp135 and the basic nitrogen present in the structure. Subsequently, the authors identified the way in which the ergoline moiety of LSD can establish aromatic contacts with Phe340 and Phe341, and how the nitrogen in the indole group forms a hydrogen bond with Gly221. Another component capable of forming interactions is the diethylamide moiety, which, thanks to one ethyl group, forms non-polar contacts with Leu132 and Trp131, while the other ethyl group interfaces with Leu362. Further interactions detected by the authors are Val136, Ser139, Leu209, Phe217, Ser222, Ala225, Trp337, and Asn344 (Figure 17A,B).

As a result of this work, a 3D structure has been deposited in the PDB (5TVN) [234]. Other structures of 5-HT2B-LSD complex are also present in the database, such as PDB ID: 7SRQ [194].

**Figure 18:**
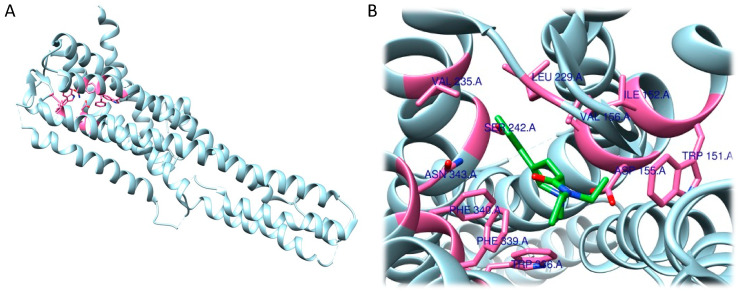
Three-dimensional model depicting 5-HT2A receptor (PDB ID 6WGT) (**A**). (**B**) Detailed view of residues (in pink) involved in interactions with LSD (green) determined by Kim et al. 2020 [70].

Given the high rate of homology between the 5-HT2A and 5-HT2B receptors, the research group of Kim et al. (2020) performed X-ray resolution of the 5-HT2A–LSD complex and noted that similarities with the homologous receptor (5-HT2B) were already present in the literature [234,236]. Additionally in this structure, the ligand inserted itself within the same orthosteric binding pocket. At the same time, the presence of the ligand allows the transducers to engage and proceed with the signalling cascade, a typical process of G-protein coupling [237]. In this case, the identified interactions involve a salt bridge between Asp155 and the nitrogen present in the molecule. In addition, hydrophobic interactions were identified with Ile152, Asn343, Leu229, Val156, Trp336, Trp151, Val235, Phe340 and Phe339. Moreover, a hydrogen bond between the nitrogen present in the indole group and Ser242 was identified (Figure 18A,B) [236]. The resulting resolved structures from this work were deposited in the PDB (6WGT) in 2020, and another group also released another structure in 2022 (7WC6) [194].

### 6.4. Therapeutic Hypotheses

The recent interest in the therapeutic effects of LSD is supported by several preclinical and clinical studies. Concerning preclinical studies, LSD has been tested to treat stress-induced anxiety-like behaviour in mice. In particular, male mice exposed to chronic restrain stress were treated daily with 30 μg/kg LSD for one week and the treatment was able to prevent the stress-induced anxiety-like behaviour, the decrease of cortical spine density and the serotonergic transmission decline [33]. Interestingly, LSD treatment did not cause any anxiolytic or anti-depressant effect in non-stressed mice. LSD has also been demonstrated to exert antidepressant effects in rodents and to promote neuroplasticity in rat cortical neurons; a single administration of LSD 0.15 mg/kg i.p. was able to significantly reduce depressive-like behaviours in rats five weeks after treatment. Indeed, by activating the TrkB receptor, mTOR and 5-HT2A signalling pathways in the prefrontal cortex, it produced an increase in dendritic arbor complexity, promote dendritic spine growth, and stimulate synapse formation, thus revealing a fast-acting, robust and persistent antidepressant effect [170,238]. Additional repeating of LSD administration (0.13 mg/kg) for 11 days was able to reverse deficits in active avoidance learning in bulbectomised rats, a model of depression [214]. Furthermore, cannabidiol (CBD) seems to exert a synergistic effect in combination with LSD on the antidepressant effect in mice, probably due to an allosteric modulation of the 5-HT2A receptor by CBD, which causes a powerful and rapid inhibition of serotonergic and glutamatergic transmission [239]. An additional potential therapeutic application of LSD concerns its positive effect on social behaviour, which can be applied in the context of mental illnesses characterized by dysfunction in social behaviour, such as autism spectrum disorders (ASD) and social anxiety disorder. It has recently been demonstrated that repeated LSD administration in adult male mice exerts a pro-social effect, measured as increased social preference and novelty in the direct social interaction and three chambers tests [240]. Furthermore, this LSD-dependent pro-social effect is flanked by alteration of the cannabinoid system in the brain, in particular decreased hippocampal levels of *N*-acylethanolamines, *N*-linoleoylethanolamine, *N*-arachidonoylethanolamine (anandamide) and *N*-docosahexaenoylethanolamine, the monoacylglycerol ½-docosahexaenoylglycerol, the prostaglandins D_2_ (PGD_2_) and F_2α_ (PGF_2α_), thromboxane 2, and kynurenine (the pathway that catabolizes 5-HT), other than by changes in the gut microbioma [241].

In healthy human subjects, neuroimaging studies have demonstrated that LSD induces increases in functional brain connectivity between the thalamus and sensory–somatomotor cortical regions, and from the thalamus to the posterior cingulate cortex [216]; on the other hand, it decreased connectivity to the temporal cortex [216]. These anatomical changes could explain the LSD-induced effect in facilitating a novel experience of the self and its environment and in reducing rigid or ruminative thinking patterns typical of psychiatric disorders [33].

In the past, several clinical trials were carried out and from these it emerged that LSD may be a useful pharmacological compound for the treatment of drug dependence, anxiety and mood disorders, especially in treatment-resistant patients [222,239,242,243]. Furthermore, many clinical studies carried out in the 60s and 70s investigated the possible use of LSD for the treatment of people with ASD and positive evidence emerged. However, this aspect needs to be investigated further with additional studies and clinical trials [29]. Different clinical trials also reported a positive effect of LSD in the context of alcohol-related disorders; indeed, a meta-analysis of randomized controlled trials concluded that a single dose of LSD is associated with a significant decrease in alcohol misuse [244].

There is only one recent clinical study investigating the potential therapeutic use of LSD. The effects of LSD were tested in a double-blind, randomized, active placebo-controlled pilot study in 12 patients suffering from anxiety associated with life-threatening diseases. LSD was administered at the doses of 20 or 200 µg during psychotherapy sessions and anxiety was measured via the State-Trait Anxiety Inventory (STAI). LSD treatment induced a significant long-lasting, up to one-year, reduction in both trait and state anxiety [222,245,246]. LSD has been reported to help patients with serious illnesses manage their emotions related to their life-threatening state of health, which reflects in decreased anxiety and depression, and increased acceptance of their potential death [246].

Safety and tolerability of repeated orally administered low doses of LSD have been tested in a clinical trial. In particular, 5 μg, 10 μg, and 20 μg LSD were administered every four days over a 21-day period to older healthy volunteers and LSD was shown to be well tolerated with no adverse events occurring [247].

On the NIH portal, 33 clinical trials are currently ongoing studying the potential therapeutic effects of LSD in the following neurological/psychiatric disorders: migraine, anxiety, major depressive disorders, ADHD and attention deficit disorder, addiction, alcohol use disorder (AUD), bipolar disorder, methamphetamine dependence, drug abuse, Alzheimer’s disease, Huntington’s disease, and both acute and chronic pain (www.clinicaltrials.gov, accessed on 15 December 2022).

Considering the strong evidence that has emerged in the last decades, the safe and well tolerated profile, and the current renewed interest in psychedelics for potential therapeutic use in psychiatric disorders, new research and new clinical studies on LSD as a pharmacological tool are needed.

## 7. Conclusions

A relevant part of the drug discovery and development approach is traditionally based on the identification of compounds from plant sources, as nature can provide an unmatched variety of complex molecular structures endowed with biological activities [248,249,250,251,252,253,254,255]. However, fully understanding the underlying molecular mechanisms through which such compounds exert their pharmacological role is not trivial.

In this review, we focused our attention on active constituents and derivatives from psychedelic plants, consisting of ibogaine, mescaline, N,N-dimethyltryptamine, psilocin and lysergic acid diethylamide. These compounds represent the main families of psychedelics, and, despite being very diverse from a chemical point of view, it has been shown that they have many aspects in common. Their use during religious and shamanic rites represents an example, and previous observational studies have shown that guided or voluptuary administration can show different effects. This translates into an aspect of primary relevance when considering such compounds as therapeutic options: the differing responses to psychedelics that have been responsible for the observed contradictory results and that are closely related to the environment in which the assisted administration occurs.

Another aspect shared by all the psychedelics discussed in this review, and that may represent a major pitfall for compounds to be developed as drug candidates, is the absence of a known specific target. However, it must be considered that in the context of plant extracts the net behavioural and psychiatric response appears to be due to the phytocomplex and potentiated by the context in which the compound is given. Again, this limitation should be considered for clinical translation. On the other hand, when the compounds are considered singularly, modern drug discovery tools, such as molecular pharmacology, computational studies and structural studies, can assist the medicinal chemist in characterizing and understanding the interaction of the bioactive compound with the macromolecular target. Of course, such preliminary data must be validated by adopting suitable in vitro and in vivo models.

Additionally, a crucial point which is related to both cultural and legislative aspects must not be ruled out. The inclusion of psychedelics among the substances of abuse has made, and still makes, pharmacological research on these molecules difficult. Nevertheless, the scientific community is currently looking with hope to the many clinical trials of psychedelics that have been developed for the treatment of depression, obsessive compulsive disorder, autism, neurodegenerative disorders, etc. In fact, compound repurposing is a constantly growing trend in the rediscovery of known compounds. Furthermore, given that psychopharmacology lacks new compounds, psychedelics and their derivatives may represent an alternative avenue for the development of new therapeutic options.

However, it must be pointed out that many issues need to be addressed before considering the development of the cited compounds in this context. First, purity, bioavailability and formulation aspects must be considered: as previously mentioned, variability is one of the main pitfalls observed during administration of psychedelics. Thus, standardization of the active constituents must be performed. Additionally, a second aspect concerns the protection of wild plant species. For example, mescaline-containing *Crassulaceae* grow only in the Rio Grande Valley and neighbouring deserts, and the germinated seed needs 20 years to develop into a mature plant. The role of efficient extractive and synthetic procedures is thus of primary relevance. Eventually, ideal dosage, route of administration and treatment regimen of every compound, together with pharmacokinetic aspects, must be fully outlined.

Moreover, the in vivo safety of all these treatments remains an open issue. In the clinic, randomized double-blind clinical trials conducted on a substantial number of subjects are very limited, inducing the current partial understanding of the therapeutic possibilities of these drugs. Note that the low dosage, up to micro-dosing, of psychedelics and medical-assisted care during the pharmacological treatments are inseparable requirements for appreciating the therapeutic potential of these highly discussed molecules. The scientific community must play a crucial role in the coming years to better define pharmacological action and disseminate to society the therapeutic potential, limits, safety, and risks associated with psychedelics therapy. Fundamental will be a strong cooperation with psychiatrists, clinicians, psychologists, pharmacologists, and chemists to map the future of psychedelics in medicine.

Thus, given the great interest that all these molecules are gathering, as testified by the constantly increasing number of scientific publications, it is necessary to deeply investigate, in combination with in silico biomedicine that has preclinical and clinical evidence, their efficacy and toxicological aspects.

## Data Availability

Not applicable.

## Abbreviations

AD	Alzheimer’s disease
ADHD	attention deficit hyperactivity disorder
ASD	autism spectrum disorders
AUC	area under the curve
AUD	alcohol use disorder
BDNF	brain-derived neurotrophic factor
BPD	bipolar disorder
CBD	cannabidiol
CNS	central nervous system
DMT	N,N-dimethyltryptamine
DSM-V	Diagnostic and Statistical Manual of Mental Disorders, Fifth Edition
FDA	Food and Drug Administration
fMRI	functional magnetic resonance imaging
FRA	flexible docking mode
GDNF	glial cell line-derived neurotrophic factor
5-HT	serotonin
IND	investigational new drug
iPSC	induced pluripotent stem cells
LSD	D-lysergic acid diethylamide
MADRS	Montgomery–Åsberg Depression Rating Scale
mGluR	metabotropic glutamate receptor
mTOR	mammalian target rapamycin receptor
NIH	National Institutes of Health
NGF	neural growth factor
NMDA	N-methyl-D-aspartate
NSSs	neurotransmitter sodium symporters
6-OHDA	6-hydroxydopamine
OUD	opioid use disorder
PAP	assisted psychotherapy
PD	Parkinson’s disease
PDB	protein data bank
PET	positron emission tomography
PG	prostaglandin
PTSD	post-traumatic stress disorder
RMSF	root-mean-square-fluctuation
RRA	rigid docking mode
SERT	serotonin transporter
STAI	state-trait anxiety inventory
SV2A	synaptic vesicle glycoprotein 2A
TAARs	trace amine associated receptors
TrkB	tropomyosin kinase B
VMAT2	vesicular monoamine transporter 2
VTA	ventral tegmental area
